# Clinicopathological and biological significance of mitotic centromere-associated kinesin overexpression in human gastric cancer

**DOI:** 10.1038/sj.bjc.6603905

**Published:** 2007-07-24

**Authors:** Y Nakamura, F Tanaka, N Haraguchi, K Mimori, T Matsumoto, H Inoue, K Yanaga, M Mori

**Affiliations:** 1 Department of Surgery and Molecular Oncology, Medical Institute of Bioregulation, Kyushu University, 4546 Tsurumibaru, Beppu 874-0838, Japan; 2 Core Research for Evolutional Science and Technology (CREST), Japan Science and Technology Agency (JST), 4-1-8 Honcho Kawaguchi, Saitama, Japan; 3 Department of Surgery, Jikei University School of Medicine, 3-25-8 Nishi-shinbashi, Minato-ku, Tokyo, Japan

**Keywords:** MCAK, KIF2C, gastric cancer, migration, cancer testis antigen

## Abstract

Mitotic centromere-associated kinesin (MCAK) is a microtubule (MT) depolymerase necessary for ensuring proper kinetochore MT attachment during spindle formation. To determine *MCAK* expression status and its clinicopathological significance, real-time reverse transcriptase–polymerase chain reaction was used in 65 cases of gastric cancer. *MCAK* gene expression in cancer tissue was significantly higher than expression in non-malignant tissue (*P*<0.05). Elevated *MCAK* expression was significantly associated with lymphatic invasion (*P*=0.01) and lymph node metastasis (*P*=0.04). Furthermore, patients with high *MCAK* expression had a significantly poorer survival rate than those with low *MCAK* expression (*P*=0.008). Immunohistochemical study revealed that expression of MCAK was primarily observed in cancer cells. Additionally, a gastric cancer cell line (AZ521) that stably expressed MCAK was established and used to investigate the biological effects of the *MCAK* gene. *In vitro* results showed that cells transfected with *MCAK* had a high rate of proliferation (*P*<0.001) and increased migratory ability (*P*<0.001) compared to mock-transfected cells. This study demonstrated that elevated expression of *MCAK* may be associated with lymphatic invasion, lymph node metastasis, and poor prognosis. These characteristics may be due in part to the increased proliferative and migratory ability of cells expressing MCAK.

Gastric cancer is the fifth most common malignancy and the second leading cause of cancer death in the world. Significant geographic variation exists, with high-risk areas such as Japan, Eastern Asia, and Central and South America (Terry *et al*, 2002; Sakamoto *et al*, 2004). The incidence is relatively low in the United States; however, gastric cancer is a significant cause of morbidity and mortality, with 23 000 cases per year, resulting in 12 000 annual deaths (Jemal *et al*, 2004). To improve survival rate, development of novel treatments is crucial, and new molecular targets are desirable.

Mitotic centromere-associated kinesin (MCAK), which is a member of kinesin-13, is found throughout the cell, and is especially concentrated at the centromeres, kinetochores, and spindle poles (Wordeman and Mitchison, 1995). Kinesin family (KIF) proteins with the kinesin motor catalytic domain and the coiled-coil domain are microtubule (MT)-dependent molecular motors (Brady, 1985; Vale *et al*, 1985; Hirokawa *et al*, 1989; Endow, 2003). Members of the kinesin superfamily play an important role in intracellular transport and cell division (Wittmann *et al*, 2001). The MT cytoskeleton is a dynamic structure in which the length of the MTs is tightly regulated. One regulatory mechanism is depolymerisation of MTs by motor proteins in the kinesin-13 family (Helenius *et al*, 2006). The human genome has three distinct genes encoding the following kinesin-13 family members: Kif2a (chromosome 5q12), Kif2b (chromosome 17q22), and MCAK/Kif2c (chromosome 1p34). Unlike other kinesins that transport cargo, MCAK and other kinesin-13 members catalyse MT disassembly, a key element in normal chromosome movement (Maney *et al*, 1998; Helenius *et al*, 2006). Overexpression of wild-type MCAK is known to induce depolymerisation of MTs in both mitotic and interphase cells (Kline-Smith and Walczak, 2002).

Regarding the association of MCAK with cancer, increased levels of both MCAK and the Aurora kinases have been reported to be correlated with tumorigenesis (Perou *et al*, 1999). Overexpression of MCAK causes a moderate increase in the frequency of multipolar spindles (Holmfeldt *et al*, 2004) and monopolar spindles (Kline-Smith and Walczak, 2002), which could contribute to the gain or loss of chromosomes in daughter cells. Mitotic centromere-associated kinesin mRNA was reported to be highly expressed in colon cancer tissue (Mori *et al*, 1993; Scanlan *et al*, 2002). There have been no reports on the functional analysis of MCAK expression in human gastric cancer, the second most common cancer in Japan.

In this study, we examined the clinicopathological and prognostic significance of MCAK expression in gastric cancers. We report on the overexpression of MCAK and the association of MCAK expression with lymphatic invasion, lymph node metastasis, and poor prognosis in gastric cancer.

## MATERIALS AND METHODS

### Clinical cases

#### Patients and sample collection

Primary gastric cancer specimens and matched control samples were obtained from 65 patients who underwent surgery at the Medical Institute of Bioregulation Hospital, Kyushu University. All specimens were immediately frozen in liquid nitrogen and stored at −80°C until RNA extraction. Written informed consent was obtained from all patients. Whenever possible, specimens were also prepared for immunohistochemical studies. All 65 patients were clearly identified as having gastric cancer, based on the clinicopathologic findings. No patients received chemotherapy or radiotherapy before surgery. All patients were closely followed after surgery at regular 1-month intervals. The follow-up periods ranged from 2 to 67 months, with a mean of 30 months. All data, including age, sex, serosal invasion, lymphatic invasion, lymph node metastasis, vascular invasion, liver metastasis and histology were obtained from the clinical and pathologic records.

#### Total RNA extraction

Frozen tissue specimens were homogenised in guanidium thiocyanate, and total RNA was obtained by ultracentrifugation through a cesium chloride cushion, as described previously (Mori *et al*, 1993).

#### Evaluation of MCAK expression in clinical samples

As described previously, cDNA was synthesised from 8.0 *μ*g of total RNA (Inoue *et al*, 1995). The glyceraldehyde-3-phosphate dehydrogenase (*GAPDH*) gene served as an internal control. Two gene-specific oligonucleotide primers were designed: (*MCAK*) sense, 5′-GATGGAAGCCTGCTCTAACG-3′; antisense, 5′-GAGCAGATTCCGCTTTGTTC-3′; (*GAPDH*) sense, 5′-TTGGTATCGTGGAAGGACTCA-3′; antisense, 5′-TGTCATCATATTTGGCAGGTT-3′. The following temperature profile was used: initial denaturation at 95°C for 10 min, followed by 40 cycles of denaturation at 95°C for 10 s, annealing at 66°C (60°C for *GAPDH*) for 10 s, and extension at 72°C for 10 s. PCRs were performed in a LightCyclerTM system (Roche Applied Science, Indianapolis, IN, USA) using the LightCycler FastStart DNA Master SYBR Green I Kit (Roche Applied Science), as described previously (Ogawa *et al*, 2004). All concentrations were calculated relative to the concentration of the cDNA from Human Universal Reference total RNA (Clontech, Mountain View, CA, USA), and the concentration of *MCAK* was then divided by the concentration of endogenous reference (*GAPDH*) to obtain normalised expression values (Bieche *et al*, 1998a, 1998b, 1999). Each assay was performed three times to verify the results, and the mean normalised value of mRNA expression was used for subsequent analysis.

#### Immunohistochemistry

Immunohistochemical studies of surgical specimens for MCAK expression were performed on formalin-fixed, paraffin-embedded tissues from gastric cancer patients. After deparaffinisation and blocking of tissue sections, primary goat polyclonal antibody against MCAK (Abcam, Cambridge, UK) was applied at a 1 : 100 dilution, and the antigen–antibody reaction was incubated overnight at 4°C. ENVISION reagents (ENVISION+Dual Link/HRP; Dako Cytomation, Glostrup, Denmark) were used to detect the antigen–antibody reaction. All tissue sections were counterstained with haematoxylin.

### Experimental studies

#### Cell lines

The cell lines derived from human gastric cancer cell AZ521, KATO3, MKN1, MKN7, MKN28, MKN45, MKN74, NUGC3, NUGC4, SH10TC, and the human fibrosarcoma cell HT1080 were obtained from the Cell Resource Center for Biomedical Research Institute of Development, Aging and Cancer (Tohoku University, Sendai, Japan) and maintained in RPMI-1640 medium containing 10% (v v^−1^) fetal bovine serum (FBS) and antibiotics at 37°C in a 5% humidified CO_2_ atmosphere.

#### DNA transfection

The MCAK gene expression vector (EGFP-HsMCAK) was a generous gift from Professor Linda Wordeman (University of Washington School of Medicine, Seattle, WA, USA) (Moore and Wordeman, 2004). To confirm accurate insertion into the frame of the expression vector, sequencing chemistry was performed. AZ521, a gastric cancer cell line, did not express *MCAK* mRNA among 10 gastric cancer cell lines (data not shown). Therefore, AZ521 was used for the transfection assays. The FuGENE 6 (cat. # 1815091; Roche Applied Science) transfection reagent was employed to establish permanently transfected cells, which were then selected by treatment with the antibiotic G418 (Invitrogen, Carlsbad, CA, USA) to the specific clone for *in vitro* assays, as described previously (Adrain *et al*, 1999).

#### Western blot analysis

Western blotting and immunocytochemical staining were used to confirm the expression of MCAK in the EGFP-HsMCAK- and mock-transfected cells. Total protein was extracted from samples using PRO-PREP protein extraction solution (iNtRON Biotechnology Inc., Kyungi-Do, Korea). Aliquots of total protein were applied to 10% acrylamide gradient gels. After electrophoresis, the samples were electroblotted (0.2 A, 120 min, 4°C) onto a polyvinylidene membrane (Immobilon; Millipore Inc., Bedford, MA, USA). The MCAK protein was detected with goat polyclonal antibody at a 1 : 250 dilution. The protein levels of MCAK were normalised to the level of *β*-actin protein, which was detected by a 1 : 1000 dilution of anti-*β*-actin antibody (Cytoskelton Inc., Denver, CO, USA). The blots were developed using horseradish peroxidase-conjugated anti-goat immunoglobulin (Promega Inc., Madison, WI, USA) at a dilution of 1 : 5000. Signals were detected using SuperSignal (Pierce Biotechnology Inc., Rockford, IL, USA).

#### *In vitro* assays

The 3-(4,5-dimethylthiazol-2-yl)-2,5-diphenyltetrazolium bromide (MTT) assay (Roche Diagnostics Corp. GmbH, Mannheim, Germany) was used to determine cell proliferation. Logarithmically growing MCAK- and mock-transfected AZ521 cells were seeded at 5.0 × 10^3^ cells well^−1^ in 96-well flat-bottomed microtitre plates, in a final volume of 100 *μ*l culture medium per well, and incubated in a humidified atmosphere (37°C and 5% CO_2_). After incubation, 10 *μ*l of MTT labeling reagent (final concentration 0.5 mg ml^−1^) was added to each well, and the plate was incubated for 4 h in a humidified atmosphere. Solubilisation solution (100 *μ*l) was added to each well, and the plate was incubated overnight in a humidified atmosphere. After confirmation that the purple formazan crystals were completely solubilised, the absorbance of each well was measured by a Model 550 series microplate reader (Bio-Rad Laboratories, Hercules, CA, USA), at a wavelength of 570 nm corrected to 655 nm. Each independent experiment was performed three times.

For cell cycle analysis, MCAK- and mock-transfected AZ521 cells (2.0 × 10^6^) were preincubated for 24 h in 10 ml of serum-free medium at 37°C, and were then incubated in medium containing 10% FBS (v v^−1^) for an additional 18 h at 37°C. The cells were harvested and fixed in 70% ethanol at −20°C, and then washed and resuspended in propidium iodide (PI) (BD Biosciences, San Jose, CA, USA) staining buffer containing 5 *μ*g PI and 0.25 mg RNase per millilitre PBS. DNA content was measured with an EPICS XL flow cytometer (Beckman Coulter Corp., Tokyo, Japan).

Migration assays were performed using BD Falcon FluoroBlock 8 *μ*m HTS Cell Culture Inserts for 24-well plates (cat. # 351152; BD Biosciences) to evaluate invasive cells, as described previously (Albini *et al*, 1987). Briefly, cells (5.0 × 10^4^ cells per 0.5 ml per well) were placed in the upper chamber, and the lower chamber was filled with 750 *μ*l DMEM with 10% (v v^−1^) fetal calf serum as a chemoattractant. After 24, 36, and 48 h of incubation at 37°C, insert membranes were labeled with calcein-AM reagent (Molecular Probes, Carlsbad, CA, USA). Migrating cells that had passed through the membrane to its lower surface were evaluated in a fluorescence plate reader (Perkin-Elmer, Waltham, MA, USA) at excitation/emission wavelengths of 485/530 nm. Migratory ability was calculated from the ratio of fluorescence of the MCAK- or mock-transfected AZ521 cells to the fluorescence of the invasive cells of HT1080, the human fibrosarcoma cell line that served as a control.

Anoikis assays were performed in six-well Costar Ultra Low Attachment Microplates (cat. # 3471; Corning Inc., Corning, NY, USA). Mitotic centromere-associated kinesin- or mock transfected AZ521 cells were suspended in RPMI-1640 with 10% FBS at a level of 0.5 × 10^6^ cells ml^−1^, and 2 ml of cell suspension was added to each well and incubated in the microplates for 18 h in a humidified (37°C and 5% CO_2_) incubator. Following the induction of anoikis, the cells were washed and resuspended in 0.5 ml of binding buffer, and annexin V:fluorescein isothiocyanate/PI labeling was performed in accordance with the manufacturer's protocol (BD Biosciences). Analysis was performed by FACScan (BD Biosciences). Each sample contained 1 × 10^4^ cells. The data were analysed by CellQuest software (BD Biosciences). These procedures were also performed in triplicate.

### Statistical analysis

Quantitative real-time reverse transcriptase–polymerase chain reaction (RT–PCR) data and *in vitro* transfected cell assay data were analysed by JMP 5 for Windows software (SAS Institute Inc., Cary, NC, USA). Patient's overall survival rates were calculated actuarially according to the Kaplan–Meier method (Kaplan and Meier, 1958) and were measured from the day of surgery. Differences between groups were estimated using the *χ*^2^ test, Student's *t*-test, repeated-measures ANOVA test, and the log-rank test (Mantel, 1966). Variables with a value of *P*<0.1 in univariate analysis were used in a subsequent multivariate analysis based on the Cox proportional hazards model. A probability level of 0.05 was chosen for statistical significance.

## RESULTS

### Clinical cases

#### Mitotic centromere-associated kinesin expression in clinical samples

**Figure 1:**
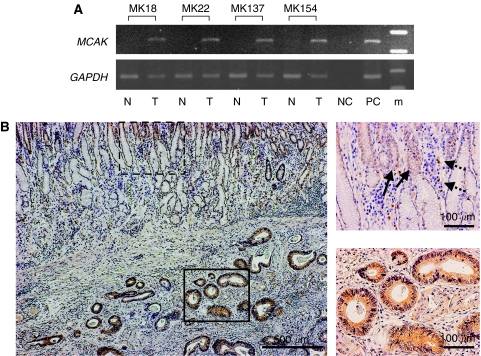
Mitotic centromere-associated kinesin mRNA expression in clinical samples. (**A**) RT–PCR analysis of MCAK in gastric cancer (T) and paired normal (N) samples obtained from four patients (patient numbers: MK 18, 22, 137, and 154). Mitotic centromere-associated kinesin mRNA expression was observed in cancer samples, but no expression was seen in normal samples. GAPDH was used as a control. m=marker; NC=negative control; PC=positive control. (**B**) Immunohistochemistry of gastric cancer surgical specimens using an antibody to MCAK. Positive staining was observed in cancer cells, but not in normal mucosal epithelium (magnification × 40 (left image)). A dotted circle indicates normal mucosa and a solid line circle indicates cancer cells. In normal mucosa (right upper image, magnification × 100), solid line arrows indicate proliferating cells of the glandular neck and dotted line arrows indicate macrophages.

Reverse transcriptase–polymerase chain reaction analysis of four representative clinical tissue samples demonstrated unequivocal *MCAK* mRNA expression in cancer samples compared with negative expression in the paired samples of adjacent normal tissue (Figure 1A). To investigate the high level of MCAK expression in the initial cancer samples, all 65 paired clinical samples of gastric cancers were then submitted for quantitative real-time RT–PCR analysis. There were 43 of 65 patients (66.2%) with a higher expression level of *MCAK* mRNA in gastric cancer tissues than in non-malignant tissues. The mean expression value of *MCAK* mRNA in cancer tissues was 0.25±0.015 (mean±s.d., normalised by *GAPDH* gene expression), which was significantly higher than the value of 0.18±0.025 in the corresponding non-malignant tissues (*P*=0.0145; Student's *t*-test). Figure 1B shows the immunohistochemical result of a representative clinical sample from a gastric cancer patient. Most of the staining is seen in the cytoplasm of cancer cells, proliferating cells of glandular neck, and macrophage, and not in stromal cells or normal epithelium. Thus, proliferating cells demonstrated high MCAK expression.

#### Clinicopathologic and prognostic analyses of gastric cancers

**Figure 2:**
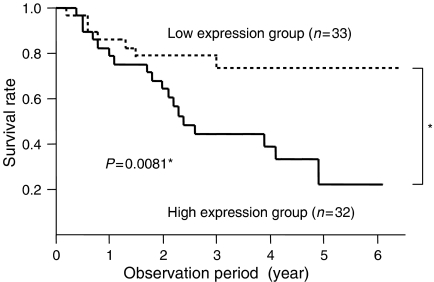
Overall survival rate of patients with gastric cancer grouped according to MCAK mRNA expression status of the tumour. Patients with high MCAK mRNA expression (*n*=32) had a significantly poorer prognosis than those with low MCAK mRNA expression (*n*=33).

In the current study, patients with less than the median *MCAK* mRNA expression ratio of cancerous to normal tissue (T/N) were assigned to the low-expression group (*n*=33), and the others were assigned to the high-expression group (*n*=32). Table 1 shows the clinicopathologic data and *MCAK* mRNA expression ratio of cancerous to normal tissue (T/N) from the 65 gastric cancer patients. The incidences of lymph node metastasis (*P*=0.04) and lymphatic invasion (*P*=0.01) were significantly higher in the high-expression group than in the low-expression group. There were no significant differences seen between the other observed clinicopathological factors that were compared between the two expression groups. Moreover, the 5-year actuarial overall survival rates in patients with a high *MCAK* expression ratio and patients with a low *MCAK* expression ratio were 45 and 79%, respectively (Figure 2). The survival difference between these two groups was statistically significant (*P*=0.008). Multivariate analysis indicated that the median expression ratio of *MCAK* was found to be an independent and significant prognostic factor for survival (OR, 1.95; CI, 1.21–3.36) (Table 2).

### Experimental studies

#### *In vitro* proliferation and cell cycle assays

**Figure 3:**
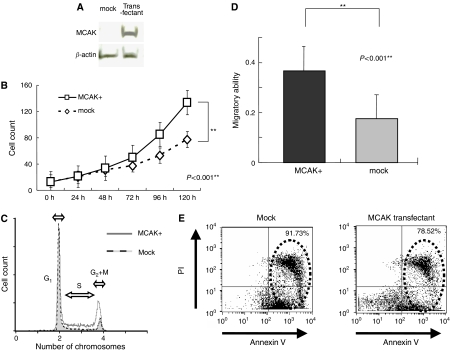
Experimental studies. (**A**) Western blotting revealed that MCAK protein was detected in transfectants but not in mock-transfected cells. *β*-Actin was used as a control. Right and left lanes show transfected and mock-transfected cells (cell line AZ521), respectively. (**B**) Growth rate of MCAK transfectants and mock-transfected cells in 10% FBS. Bar=s.d.; cell counts were greater in MCAK transfectants than in mock-transfected cells (*P*<0.001). (**C**) Cell cycle analysis of MCAK transfectants and mock-transfected cells after 24 h of serum starvation followed by 18 h serum feeding with 10% FBS. The G1-phase cell counts were unified. The S-phase fraction was greater in transfectants (44.3%) than mock-transfected cells (25.3%). (**D**) Migration assay. The migratory ability of transfectants was significantly stronger than that of mock-transfected cells (*P*<0.001). (**E**) Anoikis analysis. After anoikis induction for 18 h, the apoptosis rate was measured by annexin V and PI staining. Proportion of apoptotic MCAK-transfected cells (78.52%) was less than that of apoptotic mock-transfected cells (91.73%).

To estimate whether high *MCAK* expression affects cell growth rates, the *MCAK* gene was transfected into the gastric cancer cell line AZ521 (Figure 3A), and a proliferation assay was performed. As shown in Figure 3B, there was a significant difference in the growth rate between the *MCAK*- and the mock-transfected cells (*P*<0.001). Between original cells and mock-transduced cells, there was no significant difference in the proliferation assays (data not shown). To investigate whether the *MCAK*-transfected cells showed high proliferative activity, the cell cycle was analysed. The percentage of cells in G1-, S-, and G2+M phase of *MCAK*-transfected cells were 44.5±4.62, 40.9±11.9, and 6.43±5.83, respectively. On the other hand, the percentage of cells in G1-, S-, and G2+M phase of mock-transfected cells were 65.5±2.68, 16.3±4.71, and 17.1±8.87, respectively. The percentage of cells in S phase of *MCAK*-transfected cells was higher than the percentage of mock-transfected cells in S phase, following treatment with serum starvation for 24 h and serum refeeding for 18 h (Figure 3C). These results suggest the possibility that *MCKA* gene expression would be associated with cell proliferation because of increased cell cycling in gastric cancer cells.

#### *In vitro* migration and anoikis assays

Whether *MCAK* expression would alter the migratory ability of AZ521 gastric cancer cells was assessed in a migration assay in the condition of 24, 36, and 48 h. In all conditions, transfectants demonstrated greater motility than mock transfectants (*P*<0.001) (Figure 3D). This result corresponds to the results seen in analysed gastric cancer specimens that showed significant correlation between *MCAK* expression and invasiveness.

Anoikis is associated with cellular migration and metastatic potential. After anoikis-induced cell culture, more mock-transfected cells (91.73%) were apoptotic than *MCAK*-overexpressing cells (78.52%). *MCAK*-overexpressing cells appeared to be more resistant to anoikis induction than mock-transfected cells (Figure 3E). This anoikis assay was performed three times, and all assays revealed same results.

## DISCUSSION

Microtubules, vital components of the cytoskeleton, play important roles in mitosis, cell migration, cell signaling, and trafficking (Honore *et al*, 2005). Identification of proteins regulating the MT network of cancer cells could lead to a better understanding of regulation of tumour progression and will be helpful in improving cancer therapy. Therefore, we have investigated MCAK, which controls MT dynamics.

To clarify the aggressive behaviour of tumours with MCAK overexpression, we used *in vitro* assays to analyse the function of MCAK in cancer cells. Gastric cancer cells transfected with MCAK demonstrated higher migratory rates and greater resistance to anoikis than mock-transfected cells. It was reported that knockdown of KIF2A, which has a similar function to MCAK as an MT depolymerase, results in loss of motility in nerve cells (Homma *et al*, 2003). Thus, Kin1 kinesins, such as KIF2A and MCAK, may regulate cell motility. The results of cell motility experiments may partly explain our finding that gastric cancer patients with tumours that express high levels of MCAK had higher rates of lymphatic invasion and metastasis, and a poorer prognosis.

The regulators of MT dynamics are important for control of chromosome movement and mitosis (Gigant *et al*, 2000; Howell *et al*, 2001; Ichihara *et al*, 2001). As for these regulators in cancer cells, the catastrophe factors of MTs such as oncoprotein (Op)18/stathmin were reported to be overexpressed in a number of human malignancies, and to be associated with cancer progression and prognosis (Roos *et al*, 1993; Bieche *et al*, 1998a, 1998b; Brattsand, 2000; Chen *et al*, 2003; Moore and Wordeman, 2004; Kouzu *et al*, 2006). Op18/stathmin is involved in the cell cycle and contributes to tumorigenesis and proliferation. Suppression of Op18/stathmin expression interferes with cell cycle progression (Melhem *et al*, 1991; Jeha *et al*, 1996; Mistry and Atweh, 2002). The present study has revealed that cancer cells overexpressing MCAK, also a regulator of MT dynamics, showed high proliferative ability compared with mock-transfected cells. In addition, cell cycles of MCAK-expressing cells were faster than cell cycles of mock-transfected cells. These results may also contribute to the explanation of the aggressive behaviour of MCAK-overexpressing tumours.

MCAK has potential as a therapeutic target. The sulfoquinovosylacylglycerols (SQAGs) found in ferns and algae were reported as novel anticancer agents that inhibit DNA polymerase (Ohta *et al*, 2001). Recently, Aoki *et al* (2005) reported that the target of SQAGs was not only DNA polymerase, but also MCAK. Some SQAGs studies have demonstrated good antitumour effects (Sahara *et al*, 1997; Matsumoto *et al*, 2002). These results, combined with our findings, suggest that this novel anticancer agent may be effective in gastric cancer patients with tumours that overexpress MCAK.

In normal human organs, MCAK gene expression is distributed only in the testis (Scanlan *et al*, 2002). Our pilot study illustrated the same result (data not shown). From SAGE analysis (www.cgap.gov), MCAK was found to be highly expressed in testis and ovaries, which contain meiotic cells and a high number of proliferating cells. Mitotic centromere-associated kinesin is expressed in other tissue types at levels considered negligible. This suggests that MCAK could be a cancer testis antigen (CTA) and could become an immunotherapy target antigen. Immunotherapy, which uses tumour-specific peptides, RNA, or tumour lysate, is now considered one of the therapeutic strategies against advanced cancer (Gilboa *et al*, 1998; Sadanaga *et al*, 2001; Shimizu *et al*, 2001). We previously reported on the expression of MAGE-1 and MAGE-3 in several cancers (Inoue *et al*, 1995), and we identified several MAGE peptides that are recognised by cytotoxic T lymphocytes (Tanaka *et al*, 1997; Fujie *et al*, 1999). On the basis of these findings, cancer-specific immunotherapy using the HLA class I restricted MAGE peptide has been used in our institution for patients with advanced cancers (Sadanaga *et al*, 2001). However, MAGE expression is restricted, and almost less than half of the tumours studied express MAGE. Thus, a novel CTA useful for immunotherapy is strongly recommended. We think that MCAK may be a candidate gene for cancer-specific immunotherapy.

It has also been reported that MCAK protein was detected from the blood of colon cancer patients by the SELEX method (Scanlan *et al*, 2002), suggesting that MCAK might be useful in diagnosis. We are now investigating whether the level of MCAK in the blood correlates with tumour progression or prognosis.

In summary, we confirmed that MCAK is a cancer-specific gene and that gastric tumours with MCAK expression were associated with lymphatic invasion, lymph node metastasis, and poor prognosis. Mitotic centromere-associated kinesin may become a molecular target of an anticancer drug or cancer-specific immunotherapy.

## Figures and Tables

**Table 1:** Relationship between MCAK status and other variables

	**MCAK expression (T/N)**		
	**High^a^ (%)**	**Low^a^ (%)**	*P*-values^*^
Number of tumours	32	33	—
Average age^a^ (years)	64.6±2.4	65.1±2.3	0.87
			
*Sex*			
Female	9 (28.1)	14 (42.4)	
Male	23 (71.9)	19 (57.6)	0.23
			
*Serosal invasion*			
+	12 (37.5)	11 (33.3)	
−	20 (62.5)	22 (66.7)	0.73
			
*Lymphatic invasion*			
+	26 (81.3)	17 (51.5)	
−	6 (18.8)	16 (48.5)	0.01
			
*Lymph node metastasis*			
+	24 (75.0)	16 (48.5)	
−	8 (25.0)	16 (48.5)	0.04
			
*Vascular invasion*			
+	9 (28.1)	4 (12.1)	
−	23 (71.9)	29 (87.9)	0.10
			
*Liver metastasis*			
+	9 (28.1)	1 (3.0)	
−	23 (71.9)	32 (97.0)	0.28
			
*Histology* b			
Well	1 (3.1)	4 (12.1)	
Mod.	12 (37.5)	6 (18.2)	
Poor	9 (28.1)	13 (39.4)	0.10

MCAK=mitotic centromere-associated kinesin; T/N=ratio of cancerous to normal tissue.

a High and low: a group of patients with the higher and the lower T/N expression of MCAK than the median T/N level, respectively.

b Well, well-differentiated adenocarcinoma; mod., moderately differentiated adenocarcinoma; poor, poorly differentiated adenocarcinoma.

* Correlation was analysed by the *χ*^2^ method. Statistical significance between patients of MCAK high and low was observed in the incidence of lymphatic permeation (0.01) as well as lymph node metastasis (0.04).

**Table 2:** Multivariate analysis for overall survival

**Variables**	**Odds ratio**	**95% CI**	***P*-values**
Lymph node metastasis	4.18	(1.87–17.86)	<0.001^‡^
Vascular invasion	0.98	(0.60–1.53)	0.947
Serosal invasion	1.54	(1.02–2.33)	0.039^‡^
MCAK expression^a^	1.95	(1.21–3.36)	0.006^‡^

CI=confidence interval; MCAK=mitotic centromere-associated kinesin.

a The hazard ratio of the higher MCAK expression compared to the lower expression for cancer death.

‡ *P*<0.05 indicates an independent and significant prognostic factor for overall survival.
